# Sine Cosine Algorithm for Elite Individual Collaborative Search and Its Application in Mechanical Optimization Designs

**DOI:** 10.3390/biomimetics8080576

**Published:** 2023-12-01

**Authors:** Junjie Tang, Lianguo Wang

**Affiliations:** College of Information Science and Technology, Gansu Agricultural University, Lanzhou 730070, China; mrtanga@163.com

**Keywords:** swarm intelligence, sine cosine algorithm, tent chaotic mapping, nonlinearity, elite individual, optimization problem, tanh parameter, m-neighborhood, global optimization, co-optimization, mechanical design optimization

## Abstract

To address the shortcomings of the sine cosine algorithm such as the low search accuracy, slow convergence speed, and easily falling into local optimality, a sine cosine algorithm for elite individual collaborative search was proposed. Firstly, tent chaotic mapping was used to initialize the population and the hyperbolic tangent function was applied non-linearly to adjust the parameters of the sine cosine algorithm, which enhanced the uniformity of population distribution and balanced the global exploration and local exploitation ability. Secondly, the search method of the sine cosine algorithm was improved by combining the search strategy of the sine cosine algorithm, the m-neighborhood locally optimal individual-guided search strategy, and the global optimal individual-guided search strategy, and, then, the three search strategies were executed alternately, which achieved collaboration, improved the convergence accuracy, and prevented the algorithm from falling into local optima. Finally, a greedy selection strategy was employed to select the best individuals for the population, which accelerated the convergence speed of the sine cosine algorithm. The simulation results illustrated that the sine cosine algorithm for elite individual collaborative search demonstrated a better optimization performance than the sine cosine algorithm, the other improved sine cosine algorithms, the other chaos-based algorithms, and other intelligent optimization algorithms. In addition, the feasibility and applicability of the sine cosine algorithm for elite individual collaborative search were further demonstrated by two mechanical optimization design experiments.

## 1. Introduction

Most of the optimization problems that exist today are inherently NP-hard and difficult to solve using basic mathematical methods. Many real-world optimization problems are also represented figuratively, and the complexity of the problems faced to be solved is increasing. Therefore, the excellent performance of swarm intelligence optimization algorithms for finding optimal values in specific systems and problems has attracted many experts and scholars to conduct research in this area.

In recent years, the proposed swarm intelligence optimization algorithms include the whale optimization algorithm (WOA) [1], grey wolf optimization algorithm [2], and Harris hawks optimization algorithm [3], as well as the salp swarm algorithm [4], Olympic optimization algorithm [5], sand cat swarm optimization algorithm [6,7], and quadratic interpolation optimization algorithm [8]. The above algorithms not only enrich the research fields, but also solve many practical problems in real life, such as engineering optimization [9,10], shop floor scheduling [11], intelligent transportation [12], software module clustering [13], wireless sensor networks [14,15], intelligent agriculture [16], EDM molding process parameters [17], centrifugal pump design [18], logistics and transport [19], etc. Many optimization problems have resulted in substantial contributions to other research fields. 

However, according to the “no free lunch” theorem [20], no algorithm is perfect and can solve all problems. Therefore, it is necessary to propose different emerging algorithms or improve existing ones. We can keep trying to improve the algorithms to the point where they are close to the optimal solution, and to make them less complex, with a shorter running time and higher optimization accuracy, while maintaining a stable performance on global optimization problems and retaining the random searchability of the algorithm.

### 1.1. The Motivation

Population initialization in the SCA [21] over-relies on randomness and does not initialize the population efficiently. Relying only on the random initialization of the population may cause the initial population to gather in a certain spatial domain, which causes the population distribution uniformity to be poor and the searching individuals to fall into a blind search in the early stage. The algorithm is prone to fall into local optimality after many iterations, which, in turn, leads to a slow convergence. In addition, the overall iterative process of the SCA utilizes the parameter *r*_1_ for linear regulation to balance the performance of the global search and local development, and the parameter *r*_1_ is set to linearly decrease from 2 to 0 according to the number of iterations; at the beginning of the iteration, *r*_1_ is at a larger value, which is favorable for the global search, and, as the number of iterations reaches the end of the iteration period, the value of the parameter linearly decreases to a smaller value, and a local search is carried out. 

However, according to the linear search changes, the two phases can easily be unevenly coordinated, and the control parameter is in the global search phase when it is already in a rapid linear decline, and cannot perform fully, causing the algorithm global search and local development to fall into an imbalance. Finally, the search strategy of the SCA is to utilize the current individual *x_i_* to guide the goal development of the global optimal individual *P_best_*, i.e., using the current individual to cause the global optimal individual to rapidly converge, to achieve the purpose of seeking the optimal solution. However, this strategy only uses the current individual *x_i_* as a guide; although it has a strong global search ability, the convergence speed is slow and the optimization accuracy is low, and it easily falls into the local optimum and may miss the potential solutions that exist around the optimal individual.

To this end, this paper proposes the sine cosine algorithm for elite individual collaborative search (SCAEICS), which employs the tent chaos mapping strategy to initialize the population and efficiently and reasonably distribute the population to enhance the performance of the algorithm. The tent chaos mapping strategy has a wide range of applications [22], and adopting a tent chaos mapping population allows the algorithm to generate a uniformly distributed population of individuals in initializing the distribution, which is much more efficient and effective compared to the traditional method of population distribution. 

Meanwhile, the hyperbolic tangent function has shown a better performance as an excitation function in the field of machine learning. The introduction of the hyperbolic tangent function in the *r*_1_ parameter effectively balances the equilibrium state between the global search and the local exploitation. In the field of optimization, the idea of elite individuals has been shown to improve the performance of algorithms, and the application of the idea of elite individuals can be seen in the literature [23,24,25]. Combining the SCA basic search strategy, the m-neighborhood locally optimal individual-guided search strategy, and the globally optimal individual-guided search strategy effectively enhances the algorithm. The three search strategies are executed alternately to realize a collaborative search, which improves the convergence accuracy and prevents the algorithm from falling into a local optimum. 

In addition, a greedy selection strategy is employed to select the population to accelerate the convergence speed. Finally, two mechanical optimization design problems are simulated to verify the effectiveness of the proposed algorithm in this paper.

### 1.2. The Contribution

Based on the above ideas and strategies, this paper proposes a sine cosine algorithm for collaborative search of elite individuals to improve the performance of SCA. The tent chaotic mapping strategy is used in the initialization phase to enhance the uniformity of the population distribution, and the hyperbolic tangent function strategy is introduced to balance the equilibrium state between global search and local exploitation. In addition, to improve the convergence accuracy and convergence speed, and to avoid the algorithm from falling into local optimality, the idea of elite individuals is introduced, which effectively solves these problems. The performance of the improved algorithm is evaluated through 23 benchmark functions, CEC2020 functions, and 2 engineering design problems. Therefore, the main contributions of this study are summarized as follows:

(1) A sine cosine algorithm for elite individual collaborative search was proposed, and SCAEICS exhibits the faster convergence speed, higher convergence accuracy, and effective escape from local optima compared to the SCA;
(2) In the improvement process, the tent chaotic mapping strategy and the hyperbolic tangent function strategy are adopted, which effectively solve the defect of the randomness of the population distribution and balance the global search and local exploitation;
(3) In addition, the concept of the collaborative search of elite individuals is combined with SCA and used to improve the search performance of SCA;
(4) The proposed SCAEICS was validated by 23 benchmark functions, CEC2020 functions, and in two mechanical engineering optimization problems, and it outperformed the basic SCA in terms of convergence performance.

### 1.3. The Structure of Organization

The rest of the paper is organized as follows. Section 2 describes the basic principles and drawback in the analysis of SCA. Section 3 describes the improvement ideas of the elite individual collaborative search sine cosine algorithm in detail. Section 4 performs comparative experiments and analysis using 23 benchmark test functions and CEC2020 test functions. Section 5 applies the SCAEICS to the optimization of mechanical designs. Section 6 gives a discussion of the proposed approach. The last section summarizes the findings of this paper and points out the direction of the next research work.

## 2. Related Research

The sine cosine algorithm (SCA) [21] was proposed by S Mirjalili in 2016. The SCA was a new type of population intelligent optimization algorithm, which seeks the optimal solution of the population by modeling the periodic oscillations of the sine cosine function, and it has the advantages of fewer parameters, simpler structure, and easier implementation. Like most algorithms, the sine cosine algorithm still has the defects of a low optimization searching accuracy, slow convergence speed, and easily falling into the local optimal value. Therefore, researchers are working to improve the sine cosine algorithm in the following two aspects.

(1) Improve the initialization, parameter setting, and algorithmic structure of the SCA.

For example, a Q-learning embedded sine cosine algorithm (QLESCA) was proposed [26], in which the algorithm controls the SCA parameters by forming Q-tables for different individuals during operation, which effectively improves the convergence speed of the SCA. A new backbone sine cosine algorithm based on domain structure was proposed [27], which mainly introduced domain structure and Gaussian sampling learning through the backbone optimization idea in the update process of the sine cosine algorithm, effectively enhancing the population exploration ability and improving the population diversity. A doubly adaptive randomized standby enhanced sine cosine algorithm was proposed by introducing the doubly adjusted weight strategy and the randomized standby strategy [28], which balanced the weight factor between exploitation and exploration, accelerated the convergence speed, and enhanced the exploration ability. 

A improved sine cosine algorithm with Lévy flight was proposed [29] by multiplying the Lévy flight distribution with the sine cosine population individual position vector with corresponding elements, and the non-linear parameter adjustment method based on the spatial distance, which effectively enhanced the convergence accuracy and improved the convergence speed of the algorithm. A spectral feature peak identification and localization method based on the improved sine cosine algorithm was proposed [30], which improved the sine cosine algorithm by an adopted dynamic conversion probability and significantly modified the performance such as the spectral identification rate and localization accuracy.

Furthermore, the sine cosine algorithm introducing the backward learning strategy was proposed [31], which effectively solved the problem of late evolutionary stagnation and improved the global optimization performance. A sine cosine algorithm based on orthogonal parallel information was proposed [32], which increased the diversity and enhanced the global search capability of the algorithm by adopting a multiple orthogonal parallel information strategy. An improved sine cosine algorithm for text feature selection was proposed [33] mainly using individual coding and adaptive weighting strategies, which improved the classification accuracy compared with other feature-selection algorithms. A population-based sine cosine algorithm for application to economic load scheduling was proposed [34], which indicated a high performance compared to other techniques. An enhanced parallel sine cosine algorithm with a single-stage synchronous and asynchronous strategy designed for solving constrained and unconstrained problems was proposed [35], which effectively sped up the convergence of the algorithm. An improved sine cosine algorithm for solving high-dimensional global optimization problems was proposed [36], in which an inertia weight factor was introduced to modify the original algorithm formula, the Gaussian function was used to reduce the algorithm parameters nonlinearly, and 24 high-dimensional functions and other large-scale global optimization problems were used to evaluate the effectiveness of the algorithm, which showed that the modified algorithm effectively avoids falling into local optimization and accelerates the convergence speed.

(2) Complement the SCA with other algorithms.

For example, an enhanced brain storm sine cosine algorithm was proposed [37] to improve population diversity by introducing the enhanced brain storm strategy and two new individual updating strategies, which achieved an effective balance between the global search and local exploitation. Meanwhile, by introducing a cloud model strategy to adaptively adjust the control parameters, a cloud model-based sine cosine algorithm was proposed [38], and the experimental results show that the improved algorithm outperformed the original algorithm in solving global optimization problems. A sine cosine algorithm embedded with differential evolution and inertia weights was proposed [39], which embedded a differential evolution algorithm with dynamic variation, and better balanced the performance of global search and local exploitation by introducing adaptive inertia weights. A hybrid multi-objective firefly algorithm and sine cosine algorithm was proposed [40], in which the results show that the modified algorithm was effective for multi-objective optimization problems. A improved sine cosine algorithm hybridized with a particle swarm algorithm for recording lung CT images of COVID-19 infected patients was proposed [41], which had a high practicality in the field of medical image alignment.

The above algorithms use different strategies to improve the working of the SCA and the improvement is better. However, by analyzing the results, these algorithms still have some limitations. Therefore, it is necessary to continuously improve the SCA to make it more applicable to real-life practical problems and to overcome the challenging new problems brought by the development of society, which is the main motivation of the current research. Next, we will work more effectively and comprehensively.

## 3. Basic Sine Cosine Algorithm

### 3.1. Principle of the Sine Cosine Algorithm

**Figure 1:**
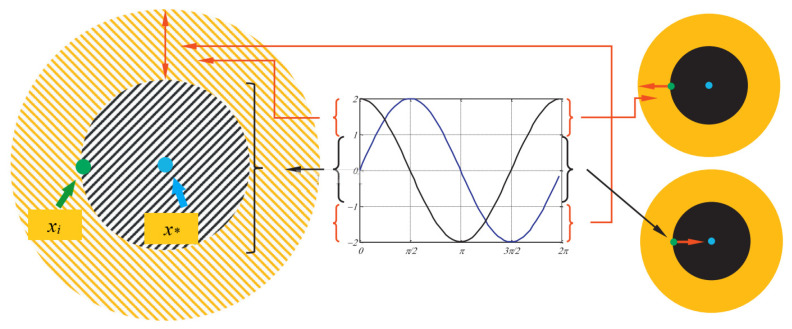
Schematic diagram of the sine cosine algorithm. The yellow striped area indicates the global search space, the black striped area indicates the local exploitation space, *x_i_* indicates the current individual, and x* indicates the target individual.

The SCA converges to the global optimal solution by modeling the periodic oscillatory nature of the sine and cosine functions by the probability progressively from the global search in the exploration phase and the local exploitation in the exploitation phase. The basic principle of the SCA is shown in Figure 1. When the position interval of the sine and cosine functions is in [2, 1] and [−2, −1], the current individual is guided to perform the global search in the exploration phase for the solution space. When the sine and cosine function location interval is at [−1, 1], the target optimal solution is exploited and the exploitation phase performance is exploited locally in the solution space. The sine and cosine functions work together in the SCA search process to complement each other, thus ultimately the SCA eventually converges to the global optimal solution.

For solving the minimization problem,

$$\min f(x)=\min f(x_{1},\;x_{2},\;\cdots ,\;x_{D})$$

where *x* is the feasible solution to the problem and *D* is the spatial dimension.

(I) Suppose the population size is *N*, the search space is *D*-dimensional, the position of the *ith* individual is denoted as *x_i_* = (*x_i_*_1_, *x_i_*_2_, …, *x_iD_*), *i* ∈ (1, 2, …, *D*), the optimal individual position in the iterative update of the *N population* is denoted as *p_best_* = (*_pbest_*_,1_, *_pbest_*_,2_, …, *_pbest_*_,*D*_), and the *ith* individual in the population updates its spatial position according to Equation (1).

(1) $$x_{i,j}^{t+1}=\left\{{}_{x_{i,j}^{t}+r_{1}\cdot \cos(r_{2})\cdot \left|r_{3}\cdot p_{best,j}^{t}-x_{i,j}^{t}\right|,\;\;\;r_{4}\geq 0.5}^{x_{i,j}^{t}+r_{1}\cdot \sin(r_{2})\cdot \left|r_{3}\cdot p_{best,j}^{t}-x_{i,j}^{t}\right|,\;\;\;r_{4}<0.5}\right.$$

where *x_i_*_,*j*_ is the *jth* dimensional component of the *ith* candidate solution and *p_best_*_,*j*_ is the *jth* dimensional component of the global optimal solution in the iteration, *j* ∈ (1, 2, …, *D*), where *r_2_* ∈ (0,2*π*), *r_3_* ∈ (0,2), and *r_4_* ∈ (0,1) are three uniformly distributed random numbers.

(II) In the SCA, the *r*_1_ parameter effectively balances the global search and local exploitation performance, gradually converging to the globally optimal solution as the number of iterations decreases linearly, and the *r*_1_ parameter is updated according to Equation (2).

(2) $$r_{1}=a-t\cdot \frac{a}{T}$$

where *a* is the *a* constant, generally taking a value of 2, *t* is the number of current iterations, and *T* is the maximum number of iterations.

### 3.2. Disadvantage Analysis of the Sine Cosine Algorithm

Good initialized populations are crucial for the swarm intelligence algorithms, and the SCA population initialization relies excessively on randomness, without efficient initialized populations. Relying only on random initialization of the population may lead to the initial population clustering in a certain spatial domain, resulting in the population distribution being poorly homogeneous and the search for individuals falling into a blind search in the early stage. The algorithm tends to fall into a local optimum after several iterations, which in turn leads to a slow convergence rate. Therefore, a tent chaotic mapping strategy was adopted in the SCAEICS to initialize the population, distribute the population efficiently and reasonably, and enhance the performance of the SCA.

Any swarm intelligence optimization algorithm needs to consider how to balance the global search and local exploitation performance. Global search explores a broader space for the population to maintain good population diversity and avoid getting trapped in local optima. The SCA uses the parameter *r*_1_ for linear regulation to balance the performance of the global search and local exploitation. The parameter *r*_1_ is set to decrease linearly from 2 to 0 according to the number of iterations, with *r*_1_ at a large value at the beginning of the iteration to facilitate global search. As the number of iterations reaches the end, the parameter value decreases linearly to a smaller value, and the local search is carried out. However, according to the linear search variation, two phases are easily unevenly coordinated, and the control parameters are in the global search phase when they are already rapidly decreasing linearly and cannot fully perform, making the global search and local development of the algorithm fall into imbalance. To solve this problem, the hyperbolic tangent function to non-linearly adjust the control parameters *r*_1_ was used in the SCAEICS.

The search strategy of the SCA adopts the current individual *x_i_* to guide global optimal individual *P_best_* to develop the objective, the current individual to the global optimal individual to quickly converge to achieve the purpose of seeking an optimal solution. However, this strategy only uses the current individual *x_i_* to guide; although it has a strong global search capability, the convergence speed is slow and the optimization accuracy is low, and it is easy to fall into the local optimum and it may miss the potential solutions around the optimal individual. Therefore, to combine the SCA search strategy, the m-neighborhood locally optimal individual-guided search strategy and the global optimal individual-guided search strategy, the SCAEICE was proposed in this paper. The three search strategies were executed alternately to achieve a collaborative search, improve the convergence accuracy, and prevent the algorithm from falling into a local optimum.

## 4. Sine Cosine Algorithm for Collaborative Search of Elite Individuals

### 4.1. Modified Strategies of the SCAEICS Algorithm

(I). Tent chaos mapping initialization

**Figure 2:**
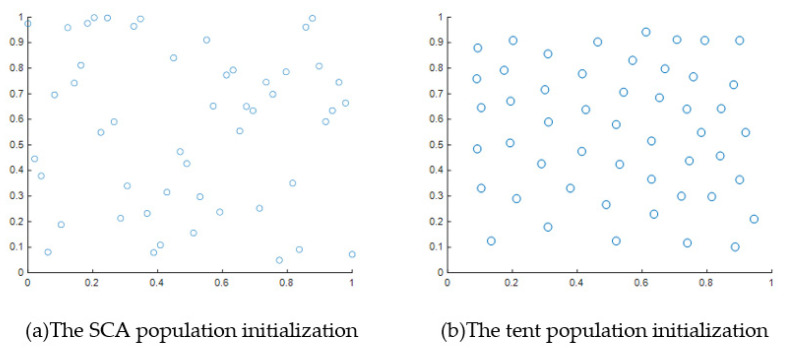
Comparison chart of population initialization (the subfigure (**a**) shows the effect of SCA population initialization, and (**b**) shows the effect of population initialization after using tent chaotic mapping).

Tent chaotic mapping [42] was used by the SCAEICS to initialize the population, so that the population was reasonably evenly distributed in the search space, the algorithm performance was improved, and the population distribution uniformity was maintained effectively. The comparison results between the tent chaos mapping initialization and random population initialization are shown in Figure 2. Tent chaos mapping not only has the characteristics of traversal uniformity and low complexity, but also preserves the initialization randomness of the SCA. The function expressions are updated according to Equation (3).

(3) $$s_{i+1,d}=\left\{{}_{u\cdot (1-s_{i,d})\;\;\;\;\geq 0.5}^{u\cdot s_{i,d}\;\;\;\;\;\;\;\;\;\;\;\;<0.5}\right.$$

where the population undergoes tent chaos mapping to generate a chaotic sequence *s_i_* = (*s_i_*, *i* = 1, 2, …, *N*), *s_i_*_,*d*_ = (*s_i_*_,*d*_, *d* = 1, 2, …, *D*), *u* ∈ (0,2); the larger the *u*, the better the chaos, and the system is in a fully chaotic state when *u* is at 2.

The mapping of the initial values of the resulting chaotic sequence in the search space yields the population *X* = (*X_i_*, *i* = 1, 2, …, *N*), *X_i_* = (*X_i_*_,*d*_, *d* = 1, 2, …, *D*), and the population individual expressions are Equation (4).

(4) $$x_{i,d}=(1+s_{i,d})\times ((ub-lb)/2)+lb$$

where *ub* and *lb* are the upper and lower bounds of the search space.

(II). The hyperbolic tangent function non-linear adjustment control parameter *r*_1_

The hyperbolic tangent [43] was adopted by the SCAEICS to improve the parameter *r*_1_. The tanh function, as an excitation function, is commonly used in the field of machine learning and has a good optimization performance for non-linear improvements. The expression of the tanh function is Equation (5).

(5) $$\mathrm{Tanh}(x)=\frac{sinh(x)}{cosh(x)}=\frac{e^{x}-e^{-x}}{e^{x}+e^{-x}}$$

**Figure 3:**
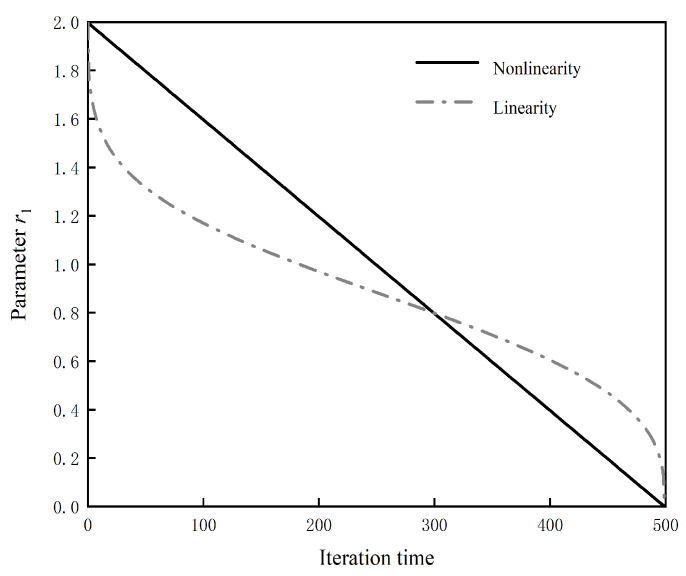
Comparison chart of parameter *r*_1_ curve.

The *sinh*(*x*) and *cosh*(*x*) are the hyperbolic *sine* functions and hyperbolic cosine functions, respectively. By introducing the tanh function into the SCAEICS, the parameters could be nonlinearly decreased, the global search capability in the early stage was enhanced, and the local development performance in the later stage was stably maintained to achieve a state of global search and local development balance, and the expression for the control parameter is improved as Equation (6).

(6) $$r_{1}=-(a_{max}-a_{min})tanh((S/T)t-S)$$

where *a_max_* and *a_min_* are the initial and termination values of parameter *a*, respectively, *t* is the current number of iterations, and *T* is the maximum number of iterations; *S* is the adjustment parameter, and the comparison of parameter *r*_1_ before and after improvement is shown in Figure 3.

(III). The elite individual collaborative search strategies

The elite individual-guided search strategy uses the best individuals of the population to guide the search process, improving the optimization accuracy and avoiding missing potential solutions and falling into local optima. The elite individual-guided search strategy is divided into the m-neighborhood locally optimal individual-guided search strategy and the global optimal individual-guided search strategy.

(IV). The m-neighborhood locally optimal individual-guided search strategy

The *M* (*m* ≤ *N*) individuals are randomly selected from the population, where the location of the optimal individual is denoted as *L_bes_*_t_ = (*l_best_*_,1_, *l_best_*_,2_, …, *l_best_*_,*D*_). The *L_best_* is the locally optimal individual in the m-neighborhood, which was searched according to Equation (7). Then, we obtain a new individual *U_i_* = (*u_i_*_,1_, *u_i_*_,2_, …, *u_i_*_,*D*_), *i* = 1, 2,…, *n.* This strategy searches in the m-neighborhood and around the locally optimal individual *L_best_*. The guidance of *L_best_* in the search is effectively utilized, considering both the search accuracy and the global search capability to prevent the algorithm from falling into the local extremes.

(7) $$u_{i,j}^{t+1}=\left\{{}_{l_{best,j}^{t}+r_{1}\cdot \cos(r_{2})\cdot \left|r_{3}\cdot l_{best,j}^{t}-x_{i,j}^{t}\right|,\;\;\;r_{4}\geq 0.5}^{l_{best,j}^{t}+r_{1}\cdot \sin(r_{2})\cdot \left|r_{3}\cdot l_{best,j}^{t}-x_{i,j}^{t}\right|,\;\;\;r_{4}<0.5}\right.$$

(V). The global optimal individual-guided search strategy

The current individual *x_i_* and the globally optimal individual *P_best_* are searched according to the search strategy in Equation (8) to obtain the new individual *U_i_*. The new individual *U_i_* was obtained by searching the current individual *x_i_* and the globally optimal individual *P_best_* according to Equation (8).

(8) $$u_{i,j}^{t+1}=\left\{{}_{p_{best,j}^{t}+r_{1}\cdot \cos(r_{2})\cdot \left|r_{3}\cdot p_{best,j}^{t}-x_{i,j}^{t}\right|,\;\;\;r_{4}\geq 0.5}^{p_{best,j}^{t}+r_{1}\cdot \sin(r_{2})\cdot \left|r_{3}\cdot p_{best,j}^{t}-x_{i,j}^{t}\right|,\;\;\;r_{4}<0.5}\right.$$

This strategy searches for the global optimal individual *P_best_* in the vicinity of the global optimal individual *P* $\left|r_{3}\cdot p_{best}-x_{i}^{t}\right|$ as the radius, in a sine or cosine manner, which not only plays the role of guiding the search by the global optimal individual *P_best_*, but also improves the optimization accuracy.

(VI). The collaborative search strategy

In the search strategy Equation (1) of the SCA, the current individual *x_i_* is searched near the current individual *x*$\left|r_{3}\cdot p_{best}-x_{i}^{t}\right|$ as the radius, in a sine or cosine manner, so that the current individual *x_i_* is close to or far from the global optimal individual.

To better balance the global and local search capabilities of the SCA, enhance the optimization accuracy, prevent falling into local extremes, and improve the quality of the optimal solution, the search strategy of the SCA, the m-neighborhood locally optimal individual-guided search strategy and the globally optimal individual-guided search strategy are combined, and the three search strategies are executed alternately to realize a collaborative search. The collaborative search strategy, which preserves the search mechanism of the SCA, makes full use of the ability of global search and prevents the algorithm from falling into local extremes. The elite individual-guided search mechanism plays the role of the *p_best_* global optimal individual and the *l_best_* local optimal individual to guide the search process and conducts a local search in the vicinity of the *p_best_* global optimal individual and the *l_best_* local optimal individual to improve the optimization accuracy and enhance the quality of the optimal solution of the algorithm. At the same time, this allows the use of the *l_best_* local optimal individual guidance, and a balance of global search ability and local search ability.

(VII). The greedy selection strategy

The new individual *u_i_* derived from the elite individual collaborative search strategy is greedily selected with the individual *x_i_*, and the better individual of the two is retained to improve the accuracy of the algorithm and accelerate the convergence speed. In Equation (9), *f*(−) is the objective function fitness value.

(9) $$x_{i}{}^{t+1}=\left\{{}_{\;\;\;\;\;\;x_{i}^{t},\;\;\;\;\;\;\;\;\;\;\;\;f(u_{i}^{t})\geq f(x_{i}{}^{t})}^{\;\;\;\;\;\;u_{i}^{t},\;\;\;\;\;\;\;\;\;\;\;\;f(u_{i}^{t})<f(x_{i}{}^{t})}\right.$$

### 4.2. Algorithm Implementation Steps

The SCAEICS algorithm is implemented in the following steps.

Step 1: Tent chaos mapping initializes the population, the population size is set to *N*, the current number of iterations *t*, the maximum number of iterations *T* of the algorithm, the spatial dimension *D*, the number of neighbors *m*, and the iteration interval *h* of the elite individual collaborative search strategy.

Step 2: Calculate the fitness of each individual and find the globally optimal individual *p_best_*.

Step 3: Calculate the control parameter *r*_1_.

Step 4: Execute the search strategy of the SCA according to Equation (1) when *t* mod 2 == 0, and turn to Step 6, otherwise execute Step 5.

Step 5: Execute the elite individual-guided search mechanism. Generate a random number *h* between [0,1], and when *h* > 0.5, execute the m-neighborhood locally optimal individual-guided search strategy according to Equation (7). Otherwise, execute the global optimal individual-guided search strategy according to Equation (8).

Step 6: Execute the greedy selection strategy according to Equation (9) and update the current individual.

Step 7: Update the global optimal individual.

Step 8: If *t* > *T*, stop the iteration and output the global optimal solution. Otherwise, *t* = *t* + 1 and move to Step 3.

#### 4.2.1. Pseudo-Code for the SCAEICS Algorithm

The pseudo-code of the SCAEICS algorithm is shown in Algorithm 1.
**Algorithm 1:** Sine cosine algorithm for the collaborative search of elite individuals (SCAEICS)Enter parameters and initialize.Set the population size *N*, use the tent chaos mapping strategy to generate the initialized population, (*x_i_*, *i* = 1, 2, …, *N*), and the maximum number of iterations *T*. Set the neighborhood individuals *m*, integer *h*, and spatial dimension *D* (where the function *f*_14_~*f*_23_ is a fixed dimension).Calculate the individual fitness value *f*(*x_i_*), *i* = 1, 2, …, *N*) and find the globally optimal individual and its location.*t* = 0;While (*t* < T) do                Identifying locally optimal individuals and their locations.         for *i* = 1 *to N do*                        Calculate the value of the control parameter *r*_1_ according to Equation (6).               if (*t* mod 2==0)                               The SCA search strategy is executed according to Equation (1).                       else if (*h* > 0.5)                     Execute the m-neighborhood locally optimal individual-guided search strategy according to Equation (7).                                 else if                                           Execute the globally optimal individual guided search strategy according to Equation (8).                end if                      end if              end if  Execute the greedy selection strategy according to Equation (9).         end for                     Updating the current optimal individual and position.*t* = *t* + 1;end while

#### 4.2.2. Flowchart of the SCAEICS Algorithm

**Figure 4:**
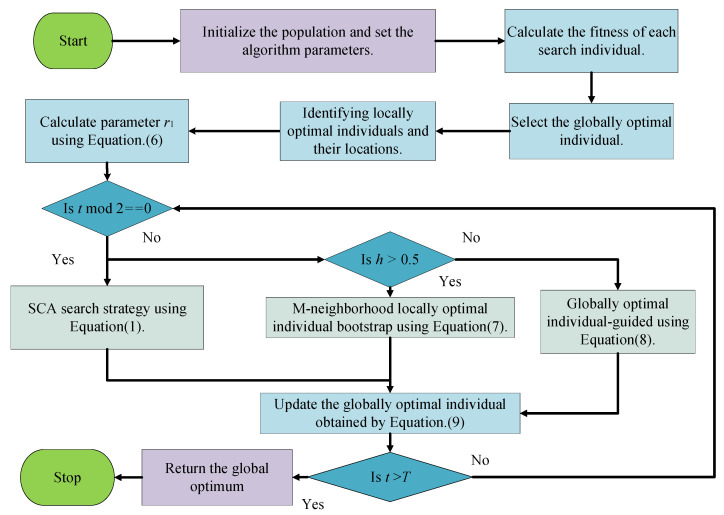
The flowchart of the proposed algorithm.

The flowchart of the SCAEICS algorithm is shown in Figure 4.

### 4.3. Analysis of Algorithm Convergence and Diversity

The advantages and disadvantages of convergence in intelligent algorithms determine the performance of the algorithm to a large degree. Therefore, in the improvement work of intelligent algorithms, many scholars have analyzed the convergence in detail. For example, an analysis of the convergence of the ABC algorithm was proposed [44] using the relationship between the algorithm variables and the general solution of the objective optimal solution update equation. 

A convergence analysis of the ABC algorithm based on von Neumann stability and convergence was proposed [45]. A convergence analysis of the PSO algorithm guided by backward learning was proposed [46]. An analysis of the convergence of the improved SCA algorithm for population diversity defined by the population center of gravity was proposed [47]. An analysis of the convergence of the improved SCA algorithm for Markov chains was proposed [48]. 

The iterative update of the SCAEICS belongs to the Markov chain of stochastic search. Therefore, based on the above literature analysis, it is clear that the SCAEICS combines the SCA search mechanism with the elite individual-guided search mechanism and converges to the globally optimal solution using a greedy selection strategy. How to balance the local exploration and global development is an important evaluation indicator for optimization algorithms, and the importance of diversity in this process is relatively important. Therefore, conducting diversity analysis on the SCAEICS is of great significance. This article refers to the diversity analysis of the SCAEICS conducted in reference [22]. Due to the use of tent chaos mapping initialization, the population distribution is uniform and a good population diversity is maintained.

## 5. Simulation Experiments

Experimental environments include an Intel(R) Core (TM) i7−10750H CPU@ 2.30 GHz, 16 GB RAM, Windows 10 operating system, and the whole algorithms were compiled and implemented using the MATLAB R 2020b simulation platform.

(I). Benchmark functions and parameter settings

To analyze the performance of the proposed algorithm, 23 benchmark functions and CEC2020 benchmark functions were used for simulation experiments, and the function expressions are detailed in the literature [49]. Other parameters of the functions are shown in Table 1 and Table 2: *f*_1_ to *f*_7_ are single-peaked dimensional functions, *f*_8_ to *f*_13_ are multi-peaked dimensional functions, and *f*_13_ to *f*_23_ are fixed dimensional multi-peaked functions. In the single-peaked dimensional function, the test dimension has one local extremum, which is used to verify the convergence speed of the algorithm and its performance, and in the fixed-dimensional multi-peaked function, the test dimension has multiple complex local extremums, which are used to verify the global search performance of the algorithm and its ability to escape from the local optimum. *F*_1_ to *F*_10_ are unimodal, multimodal, new hybrid, and composite, with significance for verifying the SCAECIS performance.

(II). Parameter settings of other algorithms involved in the following comparison

To verify the effectiveness and superiority of the SCAEICS, 23 benchmark functions and CEC2020 benchmark functions were used to compare the SCAEICS with other algorithms. To ensure fair results, the involved algorithms in the comparison were uniformly set to a population size of *N* = 30 and a maximum number of iterations *T* = 500, and each algorithm was run 30 times independently. The parameters of the SCAEICS were set to *a_max_* = 2, *a_min_* = 0, *S* = 2, *m* = 6, and *h* = 1, and the other parameters of other algorithms involved in the following comparison are shown in Table 3.

### 5.1. Comparative Analysis of the SCAEICS with the SCA and Other Intelligent Algorithms

To verify the performance of the SCAEICS, 23 benchmark functions were used to compare it with the SCA and other swarm intelligence algorithms proposed in recent years, which are the whale optimization algorithm (WOA) [1], grey wolf optimization algorithm (GWO) [2], Harris hawks algorithm (HHO) [3], and salp swarm algorithm (SSA) [4]. The parameters of the algorithms involved in the comparison are detailed above, the best values of the comparison results are bolded, and the experimental results are shown in Table 4.

As can be seen from Table 4, the SCAEICS is comparable to the SCA in experimental results; except for the optimization effect of the function *f*_16_, the other 22 functions are better than the SCA. The experimental results show that the SCAEICS has shown a significant optimization performance, for the single-peak dimension functions *f*_1_*~f*_7_, the optimization effect of four functions has a large degree, including *f*_1_, *f*_2_, *f*_3_, *f*_4_, etc. For the multi-peak dimensional functions *f*_8_ to *f*_13_, three of them, *f*_8_, *f*_9_ and *f*_10_, have reached the theoretical optimum. For the fixed multi-peak dimensional functions *f*_14_ to *f*_23_, five of them, *f*_16_, *f*_18_, *f*_21_, *f*_22_ and *f*_23_, have reached the theoretical optimum. This is because the SCAEICS retains the search strategy of the SCA and adopts a combination of the m-neighborhood local optimal individual-guided search strategy and the global optimal individual-guided search strategy, and the three search strategies are executed alternately to achieve a collaborative search, presenting a better collaborative capability, and the global search and local exploitation achieve a desirable balance effect.

To further analyze the stability and superiority of the SCAEICS, comparative experiments were conducted with other algorithms proposed in recent years, and the Wilcoxon rank sum test with a significance level of 5% was used to analyze the significant differences between the algorithms. The decision results (+/=/−) indicate the number of functions in which the compared algorithms are “better/equal/worse” than the SCAEICS, respectively. The Wilcoxon rank sum test results show that the SCAEICS outperformed the SCA on 21 functions, the WOA on 19 functions, the GWO on 18 functions, the HHO on 13 functions, and the SSA on 18 functions.

**Figure 5:**
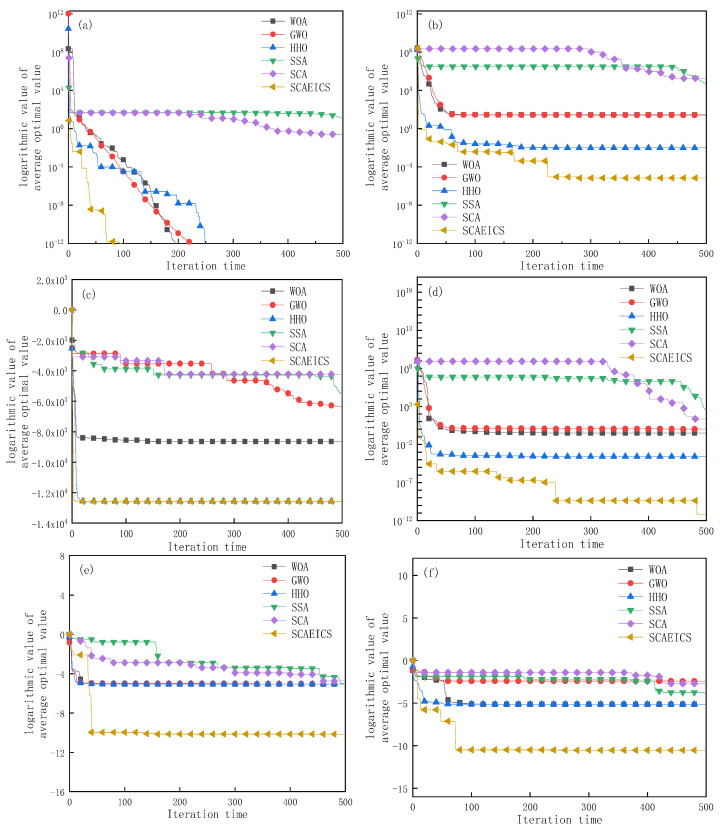
Plot of average convergence curves: (**a**) *f*_2_; (**b**) *f*_5_; (**c**) *f*_8_; (**d**) *f*_13_; (**e**) *f*_21_; (**f**) *f*_23_.

The data in Figure 5 are from the convergence graphs plotted for 30 runs of six selected functions, *f*_2_, *f*_5_, *f*_8_, *f*_13_, *f*_21_, and *f*_23_, from the Table 4 comparison experiment. The vertical co-ordinate is represented by the logarithm of the average optimal value of the function, and the horizontal co-ordinate is the number of iterations.

From the analysis in Figure 5, it can be seen that among the single-peaked dimensional functions: For *f*_2_, the SCAEICS converges significantly faster and has a higher optimization accuracy compared to the other five algorithms. For *f*_5_, the SCAEICS converges significantly faster than the other five algorithms, starting to converge at about 230 iterations until convergence to the global optimal solution. Among the multi-peaked dimensional functions, the *f*_8_ function has difficulty in finding an optimum due to its high number of local optima. For *f*_8_, the SCAEICS converges slightly faster compared to the HHO, starting to converge at about 10 iterations until it converges to the global optimum. For *f*_13_, the SCAEICS converges faster than the other five algorithms at the beginning of the iteration and starts to converge at about 250 iterations until it converges to the global optimum. 

In the fixed multi-peaked dimensional function, for *f*_21_, although the convergence rate is slightly lower than that of the HHO at the beginning of the iteration compared to the other five algorithms, the SCAEICS starts to converge ahead of the other five algorithms until the theoretical optimum at about 50 iterations. For *f*_23_, the SCAEICS shows significant outperformance in the early iterations compared to the other five algorithms and starts to converge to the theoretical optimum at about 70 iterations. The convergence curves demonstrate the remarkable optimization-seeking performance of the SCAEICS, which reaches global optimality on most functions and has a higher convergence accuracy compared to the SCA and the other algorithms.

In the study of intelligent optimization algorithms, the setting of different dimensions has a certain influence on the experimental results. Therefore, to verify the SCAEICS can maintain a stable performance while searching the optimum in different dimensions, this paper extends the dimensions of the *f*_1_*~f*_13_ functions to 60 and 100 dimensions, with the other parameters unchanged, and uses the Wilcoxon rank sum test with a significance level of 5% to analyze the significant differences between different dimensions of the SCAEICS in the function optimization problem, and the experimental results are shown in Table 5 and Table 6.

As can be seen from Table 5, in the 60-dimensional comparative experimental results, the SCAEICS outperforms the other algorithms for the remaining 10 functions, except for 3 functions including *f*_9_, *f*_10_, and *f*_11_, whose optimization results are comparable with the other algorithms. As can be seen from Table 6, in the results of the comparative experiments in 100 dimensions, the SCAEICS outperforms the other algorithms except for 3 functions including *f*_9_, *f*_10_, and *f*_11_, whose optimization effects are comparable with those of the other algorithms, and the other 10 functions. The analysis of the results shows that the SCAEICS has a better optimization effect in the same dimension and has a stable optimization-seeking performance in different dimensions.

To analyze the stability and superiority of the SCAEICS in different dimensions, the Wilcoxon rank sum test with a significance level of 5% was used to analyze the significant differences between the SCAEICS. The decision results (+/=/−) indicate the number of functions in which the comparison algorithm is “better than/equal to/worse than” the SCAEICS, respectively. The Wilcoxon rank sum test shows that the SCAEICS outperformed the HHO on 9 functions and the SCA, the WOA, the GWO, and the SSA on 13 functions in the 60-dimensional comparison experiment.

In the 100-dimensional comparative experimental results, the SCAEICS outperformed the SCA on 13 functions, the WOA on 12 functions, the GWO on 13 functions, the HHO on 8 functions, and the SSA on 13 functions. The results show that the SCAEICS maintains a stable performance in the search for excellence in different dimensions.

### 5.2. Comparative Analysis of the SCAEICS with Other Improved Algorithms

To further validate the performance of the SCAEICS, 23 benchmark functions were used to compare it with other improved SCA algorithms: an alternating sine cosine algorithm based on an elite chaotic search strategy (COSCA) [48], a memory-guided sine cosine algorithm for global optimization (MGSCA) [50], a sine cosine algorithm based on differential evolution (SCADE) [51], and a cloud model-based sine cosine algorithm (CSCA) [38]. The comparative analysis was carried out and the Wilcoxon rank sum test with a significance level of 5% was used to analyze the significant differences of the SCAEICS, the algorithm parameters are detailed above, where the SCADE data were taken from the literature and the rest of the data were obtained from the experiments, the best values of the comparison results are bolded, the experimental results are shown in Table 7.

As can be seen from Table 7, the experimental results of comparing the SCAEICS with other improved algorithms in the SCAEICS obtained significantly better optimization results than the other improved algorithms for 18 functions including *f*_1_~*f*_13_, *f*_16_, *f*_20_~*f*_23_, and several functions achieved theoretical optimal values. To analyze the stability and superiority of the SCAEICS more precisely, the Wilcoxon rank sum test with a significance level of 5% was used to further analyze the significant differences of the SCAEICS, and the meanings of the symbols in the decision results are shown above. The Wilcoxon rank sum test shows that the SCAEICS outperforms the COSCA algorithm on 15 functions, the SCADE algorithm on 13 functions, the MGSCA algorithm on 18 functions, and the CSCA algorithm on 19 functions.

In summary, compared to the other improved algorithms, the SCAEICS has a stronger optimization accuracy, faster convergence, which indicates the modified algorithm is able to handle different conditions of the optimization search problem.

### 5.3. Comparison and Analysis of the SCAEICS with Other Chaos-Based Algorithms

To further validate the performance of the SCAEICS, CEC2020 benchmark functions were used to compare it with the other chaos-based algorithms: the elite chaotic manta ray algorithm integrated with chaotic initialization and opposition-based learning (CMRFO) [52], chaos marine predators algorithm (CMPA) [49], sine cosine algorithm (SCA) [21]. The comparative analysis was carried out and the Wilcoxon rank sum test with a significance level of 5% was used to analyze the significant differences of the SCAEICS. The algorithm parameters are detailed above, the best values of the comparison results are bolded, and the experimental results are shown in Table 8.

As can be seen from Table 8, the SCAEICS is compared with other chaos-based algorithms in the experimental results. Compared with the CMRFO, the SCAEICS obtains significantly better optimization results on six functions including *F*_2_, *F*_5_, *F*_6_, *F*_8_, *F*_9_, *F*_10_, three functions including *F*_3_, *F*_4_, *F*_7_ are comparable to the CMRFO and the remaining function is inferior to the CMRFO. Compared with the CMPA, the SCAEICS obtains significantly better optimization on three functions including *F*_1_, *F*_3_, *F*_4_, four functions including *F*_6_, *F*_8_, *F*_9_, *F*_10_ are comparable to the CMPA, and the remaining three functions are inferior to the CMPA. Compared with the SCA, the SCAEICS obtains significantly better optimization on 10 functions than the SCA.

To analyze the stability and superiority of the SCAEICS more precisely, the Wilcoxon rank sum test with a significance level of 5% was used to further analyze the significant differences of the SCAEICS, and the meanings of the symbols in the decision results are shown above. The Wilcoxon rank sum test shows that the SCAEICS outperforms the CMRFO algorithm on 6 functions, the CMPA algorithm on 3 functions, and the SCA algorithm on 10 functions.

In summary, compared to other chaos-based algorithms, the SCAEICS has a stronger optimization accuracy and a faster convergence, which indicates the modified algorithm is able to handle different conditions of the optimization search problem.

### 5.4. Analysis of Important Parameters

The setting of the local population size *m* of the locally optimal individual *l_best_* is one of the more important parameters in the SCAEICS and has a large impact on the optimization accuracy of solving different problem functions. Taking a larger number in the local population size affects the local optimization performance and risks premature convergence of the algorithm, while conversely taking a smaller number has too little impact to achieve the desired algorithm performance. Therefore, the number of local populations *m* is set to 4/5/6/7, respectively, and the effect of the number of local populations on the performance of the algorithm is tested by simulation experiments. In total, 9 of the 23 tested functions, including the single-peaked dimension functions *f*_1_, *f*_3_, *f*_6_, the multi-peaked dimension function *f*_8_, *f*_13_, and the fixed multi-peaked dimension functions *f*_17_, *f*_19_, *f*_21_, *f*_23_, were used for the experiments, and the significant difference of the experimental results was indicated by the Friedman test with a significance level *α* of 5%. A *p*-value greater than 0.05 indicates significant differentiation in the parameter, whereas a value of less than 0.05 indicates that the parameter is not significantly different. The following results were analyzed by the professional statistical software IBM SPSS Statistics 21. Among the impact, the indicators are the mean best value, the standard deviation, the ranking, and the parameter value *p* of the Friedman test.

As can be seen from Table 10, the nine functions of *f*_1_, *f*_3_, *f*_6_, *f*_8_, *f*_13_, *f*_17_, *f*_19_, *f*_21_, and *f*_23_ are all less than the Friedman test value of 0.05, indicating that the algorithms corresponding to the four local population size values have significant differences, and the algorithm performance is optimal when the local population size *m* is taken as six in this paper, and the experimental test results are shown in Table 9.

### 5.5. Time Complexity Analysis

The time complexity of an algorithm has a large impact on the speed of convergence. In the SCA, it is assumed that the population size is *N*, the problem dimension is *D*, and the maximum number of iterations of the algorithm is *T*. Therefore, the time complexity of the SCA is Equation (10).

(10) O(SCA)=O(N⋅D⋅T)

In the SCAEICS, the other parameters are consistent with the SCA, and tent chaos mapping is used to initialize the population, and the time complexity of this part is *O*(*N-D*). For the m-neighborhood locally optimal individual-guided search strategy and in the global optimal individual-guided search strategy, the time complexity of this part is *O*(*N-T-h-*(*m*−1)/2) as the solution of this part is only a comparison process and therefore has less impact on the complexity of the algorithm. A greedy selection strategy is used to select individuals of the population on merit, and the time complexity of this part is *O*(*N-T*). Therefore, the time complexity of the SCAEICS is Equation (11).

(11) O(SCAEICS)≈O(N⋅D⋅T)

From the above analysis, it can be concluded that the algorithm time complexity of the SCAEICS is approximately the same compared to the SCA.

## 6. Applications

In this paper, the proposed SCAEICS was used to solve mechanical design optimization problems and to further validate the feasibility and applicability of the SCAEICS.

(I). Mechanical design optimization

Mechanical optimization design problems [53] belong to the classical problems in the field of machinery, which are mainly through the selection of design variables, objective functions, and constraints to build a mathematical model; therefore, the research application of such problems has a certain research significance for the mathematical model of this type of problem, which can generally be expressed as the following constrained optimization problem, Equation (12):

(12) $$\begin{array}{cc} & \min f(x)\\ s.t\;\;\;\; & g_{p}(x)\leq 0,\;p=1,\;2,\;\ldots ,\;j;\\ & h_{m}(x)=0,\;m=1,\;2,\;\ldots ,\;y;\\ & x_{ub}\leq x_{i}\leq x_{lb},\;i=1,\;2,\;\ldots ,\;n; \end{array}$$

where *x* is the design variable, *x = x*_1_, *x*_2_, …, *x_n_* and *f*(*x*) is the objective function; *g_p_* is the *pth* inequality constraint; *h_m_* is the *mth* equality constraint; and *x_ub_* and *x_lb_* are the upper and lower bounds of the design variable, respectively.

(II). Example of mechanical design optimization

To verify the feasibility and practicality of the SCAEICS, it is applied to the two problems of cantilever beam optimization [54] and three-bar truss optimization [55], and is compared with the whale optimization algorithm, grey wolf optimizer, Harris hawks optimization, salp swarm algorithm, sine cosine algorithm, and so on. To ensure the fairness of the experiment, the parameters of the algorithms were set as follows: the population size *N* = 30, the maximum number of iterations *T* = 1000, each algorithm was run 30 times independently and the average value was taken.

(III). Example of optimized design of a cantilever beam

The optimization objective in the cantilever beam optimization design problem is to make the mass of the rectangular section of the cantilever beam as small as possible, the mathematical expression for which is Equation (13):

(13) $$\;\left.\begin{array}{cc} & \min f(x)=0.0624\sum_{i=1}^{5}x_{i}\\ s.t\;\;\;\; & \frac{61}{x_{1}^{3}}+\frac{37}{x_{2}^{3}}+\frac{19}{x_{3}^{3}}+\frac{7}{x_{4}^{3}}+\frac{1}{x_{5}^{3}}\leq 1\\ & 0.01\leq x_{i}\leq 100\;\;\;i=1,\;2,\;3,\;4,\;5; \end{array}\right\}$$

where the minimum value of the *f*(*x*) function is the maximum mass of the rectangular section of the cantilever beam and the design variable *x_i_* denotes the height or width of the different unit beams.

The results of comparing the performance of the SCAEICS with different algorithms, for the cantilever beam optimization problem, can be seen in Table 10. The optimal solution of the SCAEICS is already better than the other algorithms for the minimum value of the function *f*(*x*). Therefore, the SCAEICS yields the optimum quality of the rectangular section of the cantilever beam compared to the other algorithms.

(IV). Modified strategies of SFLACF algorithm: Example of optimized design of a three-rod truss

The objective of the optimal design of a three-rod truss is to find the optimal three-rod truss volume by adjusting the cross-sectional area. The problem has a non-linear fitness function, three inequality constraints, and two decision optimization variables. The mathematical expression is Equation (14).

(14) $$\;\left.\begin{array}{c} \min f(x)=(2\sqrt{2x_{1}}+x_{2})\cdot l)\\ s.t\;\;\;\;\;\;g_{1}(x)=\frac{\sqrt{2x_{1}}+x_{2}}{\sqrt{2x_{1}^{2}+2x_{1}x_{2}}}\;p-\sigma \leq 0;\\ g_{1}(x)=\frac{x_{2}}{\sqrt{2x_{1}^{2}+2x_{1}x_{2}}}\;p-\sigma \leq 0;\\ g_{1}(x)=\frac{1}{\sqrt{2x_{1}^{2}+x_{1}}}\;p-\sigma \leq 0;\\ 0\leq x_{i}\leq 1,\;i=1,\;2;\\ l=100\mathrm{cm},\;p=2\mathrm{KN}/{\mathrm{cm}}^{2},\;\sigma =2\mathrm{KN}/{\mathrm{cm}}^{2} \end{array}\right\}$$

where the minimum value of the *f*(*x*) function is the optimal volume of the three-rod truss, *l* is the deflection, *p* is the buckling, *σ* is the stress constraint of the truss member, and *x*_1_ and *x*_2_ are the truss rod frame lengths on both sides for assessing the optimal cross-section.

As can be seen from Table 11, the optimal solution of the SCAEICS is 263.9055 for the three-rod truss optimization problem, which is better than the optimization results of other algorithms, indicating that the cross-sectional volume of the three-rod truss obtained by the SCAEICS is optimal.

## 7. Discussion

The elite individual collaborative search for the sine cosine algorithm proposed in this paper is a stochastic search algorithm, which searches for a suitable solution for the optimization problem in an iterative process. Therefore, a faster convergence speed, higher convergence accuracy, and escape from a local optimal solution are important indicators for evaluating the merits of the algorithm. In this paper, benchmark functions are used for performance testing between the SCAEICS and other algorithms, as well as practicality testing using two mechanical optimization design problems.

### 7.1. The Practical Managerial Significance (PMS)

In this section, we focus our discussion on the practical implications of the results of the algorithm performance comparisons; any optimization algorithm that has been proposed has certain advantages, but we have to consider the effectiveness and potential practical implications in real applications.

Therefore, we compared the SCAEICS with other algorithms on the 23 most popular test function sets with the CEC2020 test function set. It can be seen in Table 4 that the SCAEICS outperforms other optimization algorithms proposed in recent years, and it can be seen in Table 5 and Table 6 that the SCAEICS has an optimization performance applicable to different dimensions. In Table 7, it can be seen that the SCAEICS outperforms other improved SCA algorithms. In Table 8, it can be seen that the SCAEICS outperforms other chaos-based SCA algorithms. In Table 9, we have analyzed the important parameters in the algorithm in detail and obtained the values for the most applicable algorithm.

Furthermore, we have used Wilcoxon to compare the algorithms for comparison and Friedman to analyze the significance of the important parameters, and after analyzing the algorithms from a statistical point of view, we have proved that the SCAEICS has excellent optimization capabilities and is significantly competitive from a theoretical point of view.

In addition, to verify the practical application performance of the SCAEICS, two classical problems in mechanical optimal design problems are used to test the practicality: (1) the cantilever beam optimization problem, (2) the three-bar truss optimization problem. As can be seen from Table 10 and Table 11, the optimal values obtained by the SCAEICS in the cantilever beam optimization problem and the three-bar truss optimization problem are 1.3401 and 263.9055, respectively, which are better than the other algorithms involved in the comparison.

From the analysis of the above-mentioned results, it is clear that the SCAEICS has a superior performance and robustness and has the potential to solve problems existing in other areas. Therefore, the SCAEICS can be effectively applied to specific scenarios in mechanical engineering, road and bridge, agriculture, and water-conservancy construction, as well as other mechanical design fields.

### 7.2. Open Research Questions (ORQ)

In single-objective optimization problems, the SCAEICS proposed in this paper is significantly competitive. However, multi-objective problems still deserve attention in the field of optimization and the next research will focus on multi-objective optimization problems and solving more practical problems.

Next, researchers can focus on more practical problems such as shop-floor scheduling problems, image processing, text classification, transportation problems, logistic scheduling problems, agricultural water problems, other mechanical optimization design problems, and hyper-parametric optimization problems faced for machine learning and deep learning. Finally, interested researchers can further analyze its performance by improving the method.

## 8. Conclusions

To improve the optimization performance of the SCA, the problems of slow convergence, low accuracy of the search for excellence, and the tendency to fall into local optimality were addressed. A sine cosine algorithm for the collaborative search of elite individuals was proposed, with the following main improvements in the work.

(1). Tent chaos mapping was used to initialize the population with a hyperbolic tangent function non-linearly adjusting the control parameter *r*_1_, so that the population was uniformly distributed, enhancing the uniformity of population distribution and balancing the global search and local exploitation performance.

(2). By combining the search strategy of the SCA, the m-neighborhood locally optimal individual-guided search strategy, and the global optimal individual-guided search strategy, the search method of the original algorithm was improved. The above three search strategies were executed alternately to achieve a collaborative search, which effectively improved the convergence accuracy and prevented the algorithm from falling into a local optimum.

(3). A greedy selection strategy was used to select individuals of the population on merit to speed up convergence.

(4). Simulation experiments on 23 basic test functions and CEC2020 functions were conducted to compare the sine cosine algorithm for the collaborative search of elite individuals (SCAEICS) with the SCA, other improved SCA, other chaos-based algorithms, and other intelligent optimization algorithms, and the experimental results show that the SCAEICS had a better optimization performance.

(5). The feasibility and applicability of the SCAEICS were further verified by optimizing two example problems in mechanical design, which could provide new ideas for research in the field of mechanical design.

For future work, we plan to test the improved algorithm using a more novel set of CEC test functions. In addition, we are going to apply the improved algorithm to agricultural water resources for practical applications.

## Figures and Tables

**Table 1:** Basic test functions.

Functions	Function Name	Dimensionality	Search Space	Theoretical Optimal Value
*f*_1_	Sphere	30	[−100,100]	0
*f*_2_	Schwefel 2.22	30	[−10,10]	0
*f*_3_	Schwefel 1.2	30	[−100,100]	0
*f*_4_	Schwefel 2.21	30	[−100,100]	0
*f*_5_	Rosenbrock	30	[−30,30]	0
*f*_6_	Step	30	[−100,100]	0
*f*_7_	quarticWN	30	[−1.28,1.28]	0
*f*_8_	Schwefel 2.26	30	[−500,500]	−12,569.48
*f*_9_	Rastrigin	30	[−5.12,5.12]	0
*f*_10_	Ackley	30	[−32,32]	0
*f*_11_	Griewank	30	[−600,600]	0
*f*_12_	Penalized1	30	[−50,50]	0
*f*_13_	Penalized2	30	[−50,50]	0
*f*_14_	Shekel foxholes	2	[−65,65]	1
*f*_15_	Kowalk	4	[−5,5]	0.0003075
*f*_16_	Six_hump camel_back	2	[−5,5]	−1.0316
*f*_17_	Branin	2	[−5,5]	0.398
*f*_18_	Goldstein_price	2	[0,2]	3
*f*_19_	Hartman1	3	[0,1]	−3.86
*f*_20_	Hartman2	6	[0,1]	−3.32
*f*_21_	Sheke_5	4	[0,10]	−10.1532
*f*_22_	Sheke_7	4	[0,10]	−10.4028
*f*_23_	Sheke_10	4	[0,10]	−10.5363

**Table 2:** CEC2020 benchmark functions.

	Functions	Function Name	Dimensionality	Search Space	Theoretical Optimal Value
Unimodal Function	*F*_1_	Shifted and Rotated Bent Cigar Function	10	[−100,100]	100
Basic Functions	*F*_2_	Shifted and Rotated Schwefel’s Function	10	[−100,100]	1100
	*F*_3_	Shifted and Rotated Lunacek bi-Rastrigin Function	10	[−100,100]	700
	*F*_4_	Expanded Rosenbrock’s plus Griewangk’s Function	10	[−100,100]	1900
Hybri Functions	*F*_5_	Hybrid Function 1	10	[−100,100]	1700
	*F*_6_	Hybrid Function 2	10	[−100,100]	1600
	*F*_7_	Hybrid Function 3	10	[−100,100]	2100
Composition Functions	*F*_8_	Composition Function 1	10	[−100,100]	2200
	*F*_9_	Composition Function 2	10	[−100,100]	2400
	*F*_10_	Composition Function 3	10	[−100,100]	2500

**Table 3:** Table of parameter settings for the participation comparison algorithm.

Algorithms	Parameter Settings
WOA	*a* ∈ [0,2]; b = 1; *l* ∈ [−2,1]
GWO	*a* ∈ [0,2], *r*_1_ ∈ [0,1]; *r*_2_ ∈ [0,1]
HHO	*E*_0_ ∈ [−1,1]; *E* ∈ [0,1]; *r* ∈ [0,1]; *u* ∈ [0,1]; *v* ∈ [0,1]; *β* =1.5
SSA	*C*_1_ ∈ [2,0]; *C*_2_ ∈ [0,1]; *C*_3_ ∈ [0,1]
SCA	*r*_2_ ∈ [0,2π]; *r*_3_ ∈ [−2,2]; *r*_4_ ∈ [0,1]; a = 2
COSCA	*η* = 1; *a_start_* = 1; *a_end_* = 0; pr = 0.1
SCADE	*n_lim_* = 50; cr = 0.3; *k_max_* = 3; *h* = 10; *δ*^2^*_max_* = 0.6; *δ*^2^*_min_* = 0.0001; *a* = 2
MGSCA	*r*_2_ ∈ [0,2π]; *r*_3_ ∈ [−2,2]; *r*_4_ ∈ [0,1]; a = 2
CSCA	*r*_2_ ∈ [0,2π]; *r*_3_ ∈ [−2,2]; *r*_4_ ∈ [0,1]
CMRFO	*S* ∈ 2, *p* ∈ 0.1
CMPA	*r* ∈ [0,1], *p* ∈ 0.5, *v* ∈ 0.1, *u* ∈ [0,1], *FADs* ∈ 0.2

**Table 4:** Comparison of the SCAEICS with the SCA and other intelligent algorithms.

Function	Evaluation Criterion	WOA	GWO	HHO	SSA	SCA	SCAEICS
*f*_1_	Ave	2.02 × 10^−72^	1.39 × 10^−27^	3.95 × 10^−96^	1.70 × 10^−7^	8.10 × 10^1^	9.72 × 10^−232^
	Std	1.09 × 10^−71^	1.59 × 10^−27^	1.66 × 10^−95^	2.11 × 10^−7^	1.15 × 10^2^	0
*f*_2_	Ave	2.16 × 10^−50^	1.37 × 10^−16^	9.15 × 10^−51^	1.85	1.76 × 10^−1^	3.10 × 10^−123^
	Std	1.10 × 10^−49^	8.53 × 10^−17^	3.99 × 10^−50^	1.59	2.57 × 10^−1^	1.69 × 10^−122^
*f*_3_	Ave	4.47 × 10^4^	3.16 × 10^−5^	2.85 × 10^−80^	1.57 × 10^3^	1.42 × 10^4^	3.71 × 10^−230^
	Std	1.53 × 10^4^	1.44 × 10^−4^	1.20 × 10^−79^	1.15 × 10^3^	7.42 × 10^3^	0
*f*_4_	Ave	4.64 × 10^1^	9.54 × 10^−7^	2.51 × 10^−50^	1.06 × 10^1^	4.80 × 10^1^	1.45 × 10^−124^
	Std	2.86 × 10^1^	1.20 × 10^−6^	7.44 × 10^−50^	3.62	1.01 × 10^1^	7.73 × 10^−124^
*f*_5_	Ave	2.80 × 10^1^	2.72 × 10^1^	1.19 × 10^−2^	1.13 × 10^2^	3.43 × 10^5^	3.29 × 10^−8^
	Std	4.50 × 10^−1^	7.31 × 10^−1^	1.69 × 10^−2^	1.41 × 10^2^	5.99 × 10^5^	3.77 × 10^−8^
*f*_6_	Ave	3.17 × 10^−1^	7.43 × 10^−1^	9.25 × 10^−5^	2.55 × 10^−7^	1.04 × 10^2^	2.62 × 10^−10^
	Std	1.82 × 10^−1^	4.07 × 10^−1^	1.73 × 10^−4^	3.82 × 10^−7^	1.45 × 10^2^	1.12 × 10^−9^
*f*_7_	Ave	3.10 × 10^−3^	2.06 × 10^−3^	1.66 × 10^−4^	1.68 × 10^−1^	3.02 × 10^−1^	7.65 × 10^−6^
	Std	4.58 × 10^−3^	1.36 × 10^−3^	1.75 × 10^−4^	5.77 × 10^−2^	4.11 × 10^−1^	7.67 × 10^−6^
*f*_8_	Ave	−10,672.1305	−6090.7165	−12,554.3583	−6374.5963	−3785.7654	−12,569.4865
	Std	1704.9911	887.893	76.2766	743.4421	273.8353	0.00029551
*f*_9_	Ave	0	2.30	0	6.18 × 10^1^	5.28 × 10^1^	0
	Std	0	5.34	0	1.61 × 10^1^	4.14 × 10^1^	0
*f*_10_	Ave	4.44 × 10^−15^	1.03 × 10^−13^	8.88 × 10^−16^	2.57	1.62 × 10^1^	8.88 × 10^−16^
	Std	2.09 × 10^−15^	2.02 × 10^−14^	0	8.19 × 10^−1^	7.34	0
*f*_11_	Ave	1.11 × 10^−2^	6.80 × 10^−3^	0	1.57 × 10^−2^	1.60	0
	Std	6.07 × 10^−2^	1.31 × 10^−2^	0	1.32 × 10^−2^	6.49 × 10^−1^	0
*f*_12_	Ave	1.71 × 10^−2^	4.55 × 10^−2^	6.33 × 10^−6^	6.64	6.12 × 10^5^	1.45 × 10^−9^
	Std	7.51 × 10^−3^	2.28 × 10^−2^	8.21 × 10^−6^	2.95	1.86 × 10^6^	2.97 × 10^−9^
*f*_13_	Ave	4.94 × 10^−1^	7.19 × 10^−1^	1.02 × 10^−4^	2.00 × 10^1^	1.44 × 10^6^	1.69 × 10^−10^
	Std	2.51 × 10^−1^	2.21 × 10^−1^	1.19 × 10^−4^	1.64 × 10^1^	2.02 × 10^6^	3.02 × 10^−10^
*f*_14_	Ave	2.57	4.98	1.33	1.16	1.33	9.98 × 10^−1^
	Std	2.99	4.53	9.47 × 10^−1^	4.58 × 10^−1^	7.50 × 10^−1^	1.06 × 10^−6^
*f*_15_	Ave	0.00071818	0.0024681	0.00034688	0.00099878	0.001211	0.00033444
	Std	0.00054231	0.0060734	3.251× 10^−5^	0.00028419	0.00034186	1.84 × 10^−4^
*f*_16_	Ave	−1.0316	−1.0316	−1.0316	−1.0316	−1.0316	−1.0316
	Std	8.63 × 10^−10^	2.82 × 10^−8^	8.49 × 10^−10^	3.06 × 10^−14^	9.52 × 10^−5^	4.45 × 10^−2^
*f*_17_	Ave	0.39789	0.39789	0.39789	0.39789	0.4016	0.39789
	Std	1.22 × 10^−5^	2.14 × 10^−3^	7.01 × 10^−6^	2.16 × 10^−3^	2.20 × 10^−3^	8.47 × 10^−5^
*f*_18_	Ave	3.0001	3	3	3.0005	3.0003	3
	Std	4.93	5.05 × 10^−5^	6.38 × 10^−7^	5.01 × 10^−4^	3.71 × 10^−4^	6.49 × 10^−7^
*f_19_*	Ave	−3.8582	−3.8615	−3.8595	−3.8626	−3.8536	−3.8633
	Std	5.88 × 10^−2^	2.72 × 10^−3^	2.70 × 10^−3^	2.59 × 10^−4^	3.10 × 10^−3^	9.69 × 10^−3^
*f*_20_	Ave	−3.1983	−3.2541	−3.133	−3.2072	−2.8409	−3.3263
	Std	9.35 × 10^−2^	7.38 × 10^−2^	1.01 × 10^−1^	7.48 × 10^−2^	2.46 × 10^−1^	1.66 × 10^−1^
*f*_21_	Ave	−8.4313	−8.6048	−5.0507	−8.3476	−1.8336	−10.153
	Std	2.27	2.46	1.24	3.16	1.63	1.13 × 10^−3^
*f*_22_	Ave	−8.1586	−9.6926	−5.0821	−9.4209	−3.4597	−10.4024
	Std	3.23	1.62	1.58	2.04	1.81	3.95 × 10^−4^
*f*_23_	Ave	−8.4151	−10.0841	−5.2933	−9.3126	−3.8672	−0.5362
	Std	3.14	9.79 × 10^−1^	9.83 × 10^−1^	2.40	1.03	5.24 × 10^−4^
Decision result	+/=/−	2/2/19	2/3/18	3/7/13	3/2/18	1/1/21	--

**Table 5:** Comparison of the SCAEICS with the SCA and other intelligent algorithms (*D* = 60).

Function	Evaluation Criterion	WOA	GWO	HHO	SSA	SCA	SCAEICS
*f*_1_	Ave	8.79 × 10^−71^	1.03 × 10^−27^	1.20 × 10^−94^	3.02 × 10^2^	7.82 × 10^1^	4.79 × 10^−224^
	Std	3.71 × 10^−70^	1.68 × 10^−27^	6.53 × 10^−94^	8.75 × 10^1^	1.24 × 10^2^	0
*f*_2_	Ave	1.29 × 10^−50^	9.79 × 10^−17^	2.57 × 10^−51^	1.22 × 10^1^	2.06 × 10^−1^	4.23 × 10^−127^
	Std	4.22 × 10^−50^	5.86 × 10^−17^	8.54 × 10^−51^	1.97	4.10 × 10^−1^	1.11 × 10^−126^
*f*_3_	Ave	4.33 × 10^4^	4.22 × 10^−6^	9.12 × 10^−70^	6.58 × 10^3^	1.56 × 10^4^	4.11 × 10^−234^
	Std	1.36 × 10^4^	1.08 × 10^−5^	4.99 × 10^−69^	2.85 × 10^3^	6.59 × 10^3^	0
*f*_4_	Ave	5.10 × 10^1^	6.11 × 10^−7^	2.04 × 10^−48^	1.84 × 10^1^	4.61 × 10^1^	3.40 × 10^−125^
	Std	2.94 × 10^1^	6.59 × 10^−7^	9.61 × 10^−48^	3.64	1.27 × 10^1^	1.84 × 10^−124^
*f*_5_	Ave	2.80 × 10^1^	2.68 × 10^1^	1.46 × 10^−2^	2.62 × 10^4^	5.35 × 10^5^	3.77 × 10^−8^
	Std	3.90 × 10^−1^	5.50 × 10^−1^	1.64 × 10^−2^	2.91 × 10^4^	1.70 × 10^6^	7.36 × 10^−10^
*f*_6_	Ave	3.84 × 10^−1^	8.13 × 10^−1^	9.96 × 10^−5^	3.20 × 10^2^	1.29 × 10^2^	2.98 × 10^−9^
	Std	2.72 × 10^−1^	3.82 × 10^−1^	1.44 × 10^−4^	1.14 × 10^2^	1.74 × 10^2^	5.37 × 10^−9^
*f*_7_	Ave	2.56 × 10^−3^	1.88 × 10^−3^	1.64 × 10^−4^	3.31 × 10^−1^	2.16 × 10^−1^	9.11 × 10^−5^
	Std	2.79 × 10^−3^	1.22 × 10^−3^	1.40 × 10^−4^	1.08 × 10^−1^	4.31 × 10^−1^	8.65 × 10^−5^
*f*_8_	Ave	−9016.3152	−6709.0284	−12569.3642	−6699.3213	−3794.8247	−12569.4851
	Std	1.69 × 10^3^	8.88 × 10^2^	5.88 × 10^2^	7.95 × 10^2^	2.45 × 10^2^	7.73 × 10^−4^
*f*_9_	Ave	1.89 × 10^−15^	2.21	0	1.33 × 10^2^	5.44 × 10^1^	0
	Std	1.04 × 10^−14^	3.79	0	2.50 × 10^1^	4.35 × 10^1^	0
*f*_10_	Ave	3.85 × 10^−15^	9.94 × 10^−14^	8.88 × 10^−16^	6.37	1.42 × 10^1^	8.88 × 10^−16^
	Std	2.81 × 10^−15^	1.96 × 10^−14^	0	9.36 × 10^−1^	8.09	0
*f*_11_	Ave	5.91 × 10^−3^	4.42 × 10^−3^	0	3.66	1.81	0
	Std	3.24 × 10^−2^	8.23 × 10^−3^	0	8.74 × 10^−1^	1.10	0
*f*_12_	Ave	2.25 × 10^−2^	3.74 × 10^−2^	8.11 × 10^−6^	3.61 × 10^1^	1.94 × 10^6^	3.69 × 10^−10^
	Std	1.78 × 10^−2^	2.04 × 10^−2^	9.99 × 10^−6^	3.92 × 10^1^	9.39 × 10^6^	1.01 × 10^−9^
*f*_13_	Ave	5.56 × 10^−1^	5.72 × 10^−1^	7.79 × 10^−5^	2.73 × 10^3^	1.57 × 10^6^	7.07 × 10^−9^
	Std	2.20 × 10^−1^	1.77 × 10^−1^	7.82 × 10^−5^	1.05 × 10^4^	2.78 × 10^6^	1.21 × 10^−8^
Decision result	+/=/−	0/0/13	0/0/13	1/3/9	0/0/13	0/0/13	--

**Table 6:** Comparison of the SCAEICS with the SCA and other intelligent algorithms (*D* = 100).

Function	Evaluation Criterion	WOA	GWO	HHO	SSA	SCA	SCAEICS
*f*_1_	Ave	9.88 × 10^−74^	7.23 × 10^−28^	8.49 × 10^−96^	3.18 × 10^2^	9.23 × 10^1^	2.56 × 10^−216^
	Std	4.30 × 10^−73^	9.27 × 10^−28^	4.64 × 10^−95^	1.14 × 10^2^	1.42 × 10^2^	0
*f*_2_	Ave	2.23 × 10^−51^	1.13 × 10^−16^	7.58 × 10^−51^	1.23 × 10^1^	1.57 × 10^−1^	2.77 × 10^−123^
	Std	6.13 × 10^−51^	6.47 × 10^−17^	3.98 × 10^−50^	3.13	2.01 × 10^−1^	1.38 × 10^−122^
*f*_3_	Ave	4.72 × 10^4^	3.15 × 10^−5^	2.58 × 10^−70^	5.94 × 10^3^	1.33 × 10^4^	1.32 × 10^−238^
	Std	1.24 × 10^4^	9.29 × 10^−5^	1.41 × 10^−69^	2.46 × 10^3^	7.85 × 10^3^	0
*f*_4_	Ave	5.35 × 10^1^	6.68 × 10^−7^	5.78 × 10^−49^	1.87 × 10^1^	4.46 × 10^1^	1.28 × 10^−123^
	Std	2.48 × 10^1^	5.59 × 10^−7^	2.22 × 10^−48^	3.54	1.29 × 10^1^	6.96 × 10^−123^
*f*_5_	Ave	2.81 × 10^1^	2.69 × 10^1^	1.02 × 10^−2^	2.59 × 10^4^	2.66 × 10^5^	1.52 × 10^−7^
	Std	5.13 × 10^−1^	6.93 × 10^−1^	1.11 × 10^−2^	2.22 × 10^4^	4.40 × 10^5^	2.04 × 10^−7^
*f*_6_	Ave	3.32 × 10^−1^	7.66 × 10^−1^	1.46 × 10^−4^	3.02 × 10^2^	8.19 × 10^1^	7.83 × 10^−9^
	Std	2.07 × 10^−1^	3.71 × 10^−1^	2.17 × 10^−4^	7.90 × 10^1^	1.24 × 10^2^	1.99 × 10^−8^
*f*_7_	Ave	3.19 × 10^−3^	2.05 × 10^−3^	1.56 × 10^−4^	3.25 × 10^−1^	3.47 × 10^−1^	8.38 × 10^−5^
	Std	4.50 × 10^−3^	8.65 × 10^−4^	1.62 × 10^−4^	1.43 × 10^−1^	3.00 × 10^−1^	6.56 × 10^−5^
*f*_8_	Ave	−12507.8053	−5308.7488	−12568.5282	−5959.9149	−4434.8286	−12569.4857
	Std	1.61 × 10^3^	9.96 × 10^2^	4.08 × 10^1^	8.14 × 10^2^	2.66 × 10^2^	2.63 × 10^−4^
*f*_9_	Ave	0	2.55	0	1.30 × 10^2^	6.79 × 10^1^	0
	Std	0	3.34	0	2.07 × 10^1^	4.15 × 10^1^	0
*f*_10_	Ave	4.32 × 10^−15^	1.08 × 10^−13^	8.88 × 10^−16^	6.37	1.60 × 10^1^	8.88 × 10^−16^
	Std	2.72 × 10^−15^	2.07 × 10^−14^	0	8.91 × 10^−1^	7.21	0
*f*_11_	Ave	1.25 × 10^−2^	3.06 × 10^−3^	0	3.60	1.89	0
	Std	4.79 × 10^−2^	5.85 × 10^−3^	0	9.60 × 10^−1^	1.63	0
*f*_12_	Ave	1.86 × 10^−2^	5.16 × 10^−2^	1.14 × 10^−5^	2.27 × 10^1^	4.08 × 10^5^	8.64 × 10^−10^
	Std	1.34 × 10^−2^	3.00 × 10^−2^	1.85 × 10^−5^	1.33 × 10^1^	1.16 × 10^6^	1.97 × 10^−9^
*f*_13_	Ave	5.31 × 10^−1^	5.71 × 10^−1^	1.04 × 10^−4^	1.23 × 10^3^	1.81 × 10^6^	6.16 × 10^−9^
	Std	2.90 × 10^−1^	2.24 × 10^−1^	1.41 × 10^−4^	2.63 × 10^3^	3.87 × 10^6^	1.39 × 10^−8^
Decision result	+/=/−	0/1/12	0/0/13	1/4/8	0/0/13	0/0/13	--

**Table 7:** Comparison table of the SCAEICS with other improved algorithms.

Function	Evaluation Criterion	COSCA	SCADE	MGSCA	CSCA	SCAEICS
*f*_1_	Ave	2.44 × 10^−78^	9.58 × 10^−95^	7.62 × 10^−23^	7.49 × 10^−2^	9.72 × 10^−232^
	Std	3.21 × 10^−94^	4.92 × 10^−94^	1.67 × 10^−22^	1.93 × 10^−1^	0
*f*_2_	Ave	1.52 × 10^−44^	6.14 × 10^−63^	1.92 × 10^−17^	5.09 × 10^−7^	3.10 × 10^−123^
	Std	1.94 × 10^−60^	2.73 × 10^−62^	4.63 × 10^−17^	8.73 × 10^−7^	1.69 × 10^−122^
*f*_3_	Ave	1.78 × 10^−15^	1.93 × 10^−4^	2.80 × 10^−3^	5.83 × 10^3^	3.71 × 10^−230^
	Std	1.45 × 10^−30^	9.81 × 10^−4^	8.78 × 10^−3^	5.29 × 10^3^	0
*f*_4_	Ave	5.27 × 10^−35^	2.85 × 10^−9^	8.11 × 10^−3^	1.26 × 10^1^	1.45 × 10^−124^
	Std	1.91 × 10^−50^	1.53 × 10^−8^	2.34 × 10^−2^	9.50	7.73 × 10^−124^
*f*_5_	Ave	2.84 × 10^1^	2.69 × 10^1^	2.75 × 10^1^	4.17 × 10^3^	3.29 × 10−08
	Std	7.94 × 10^−16^	1.47 × 10^−1^	7.07 × 10^−1^	1.80 × 10^4^	3.77 × 10^−8^
*f*_6_	Ave	3.82	7.54 × 10^−5^	1.39	5.09	2.62 × 10^−10^
	Std	7.94 × 10^−16^	9.46 × 10^−5^	5.59 × 10^−1^	9.42 × 10^−1^	1.12 × 10^−9^
*f*_7_	Ave	3.21 × 10^−4^	8.44 × 10^−3^	3.87 × 10^−3^	7.74 × 10^−2^	7.65 × 10^−6^
	Std	6.06 × 10^−21^	7.37 × 10^−3^	2.65 × 10^−3^	5.34 × 10^−2^	7.67 × 10^−6^
*f*_8_	Ave	−3.31 × 10^3^	−1.20 × 10^4^	−6.36 × 10^3^	−3.37 × 10^3^	−12,569.4865
	Std	2.64 × 10^−12^	2.53 × 10^2^	6.41 × 10^2^	2.95 × 10^2^	0.00029551
*f*_9_	Ave	0	0	2.86 × 10^−1^	4.63 × 10^1^	0
	Std	0	0	8.88 × 10^−1^	4.93 × 10^1^	0
*f*_10_	Ave	2.48 × 10^−15^	2.13 × 10^−15^	7.39	2.89 × 10^−2^	8.88 × 10^−16^
	Std	7.05 × 10^−31^	1.76 × 10^−15^	9.88	7.60 × 10^−2^	0
*f*_11_	Ave	0	0	1.01 × 10^−2^	4.78 × 10^−1^	0
	Std	0	0	1.85 × 10^−2^	3.69 × 10^−1^	0
*f*_12_	Ave	3.68 × 10^−1^	3.45 × 10^−5^	1.00 × 10^−1^	9.39	1.45 × 10^−9^
	Std	1.73 × 10^−16^	1.66 × 10^−4^	4.78 × 10^−2^	4.22 × 10^1^	2.97 × 10^−9^
*f*_13_	Ave	2.04	8.13 × 10^−3^	1.46	6.35 × 10^2^	1.69 × 10^−10^
	Std	1.20 × 10^−15^	2.29 × 10^−2^	3.20 × 10^−1^	3.21 × 10^3^	3.02 × 10^−10^
*f*_14_	Ave	3.56	9.98 × 10^−1^	1.13	2.26	9.98 × 10^−1^
	Std	5.95 × 10^−16^	5.00 × 10^−16^	5.03 × 10^−1^	2.48	1.06 × 10^−6^
*f*_15_	Ave	7.87 × 10^−04^	7.52 × 10^−4^	0.0006979	0.0006694	0.00033444
	Std	7.75 × 10^−19^	1.54 × 10^−4^	3.45 × 10^−4^	2.44 × 10^−4^	1.84 × 10^−4^
*f*_16_	Ave	−1.0316	−1.0316	−1.0316	−1.0316	−1.0316
	Std	1.09 × 10^−16^	4.44 × 10^−16^	2.04 × 10^−8^	3.05 × 10^−5^	4.45 × 10^−2^
*f*_17_	Ave	0.39789	0.39789	0.39789	0.4003	0.39789
	Std	0	0	4.36 × 10^−6^	2.84 × 10^−3^	8.47 × 10^−5^
*f*_18_	Ave	3	3	3	3.0001	3
	Std	7.94 × 10^−16^	3.18 × 10^−7^	4.99 × 10^−6^	1.19 × 10^−4^	6.49 × 10^−7^
*f*_19_	Ave	−3.8589	−3.8628	−3.8585	−3.8572	−3.8633
	Std	1.39 × 10^−15^	7.64 × 10^−13^	3.85 × 10^−3^	3.33 × 10^−3^	9.69 × 10^−3^
*f*_20_	Ave	−3.1561	−3.3119	−3.1134	−3.1229	−3.3263
	Std	9.93 × 10^−16^	3.05 × 10^−2^	1.80 × 10^−1^	7.41 × 10^−2^	1.66 × 10^−1^
*f*_21_	Ave	−9.8534	−9.7526	−7.3713	−4.2256	−10.153
	Std	6.35 × 10^−15^	8.91 × 10^−1^	2.91	1.14	1.13 × 10^−3^
*f*_22_	Ave	−10.3208	−10.4029	−8.3904	−4.4632	−10.4024
	Std	4.36 × 10^−15^	1.45	3.21	8.68 × 10^−1^	3.95 × 10^−4^
*f*_23_	Ave	−10.4821	−10.5364	−8.7732	−4.5667	−10.5362
	Std	3.97 × 10^−15^	3.45 × 10^−14^	3.04	1.34	5.24 × 10^−4^
Decision result	+/=/−	2/6/15	2/8/13	2/3/18	2/2/19	--

**Table 8:** Comparison table of the SCAEICS with other chaos-based algorithms.

Function	Evaluation Criterion	CMRFO	CMPA	SCA	SCAEICS
*F*_1_	Ave	2.56 × 10^3^	1.732 × 10^6^	5.45 × 10^10^	1.35 × 10^4^
	Std	7.12 × 10^6^	1.178 × 10^6^	6.69 × 10^19^	3.79 × 10^5^
*F*_2_	Ave	8.57 × 10^3^	2.191 × 10^3^	1.52 × 10^4^	4.30 × 10^3^
	Std	9.48 × 10^5^	2.191 × 10^3^	1.80 × 10^5^	2.18 × 10^2^
*F*_3_	Ave	1.51 × 10^3^	2.191 × 10^3^	1.76 × 10^3^	1.67 × 10^3^
	Std	6.04 × 10^4^	2.191 × 10^3^	9.22 × 10^3^	1.98 × 10^3^
*F*_4_	Ave	1.90 × 10^3^	2.191 × 10^3^	2.04 × 10^3^	1.90 × 10^3^
	Std	0	6.347 × 10^1^	1.75 × 10^4^	1.62
*F*_5_	Ave	4.10 × 10^5^	2.066 × 10^3^	7.65 × 10^7^	2.94 × 10^3^
	Std	4.32 × 10^10^	8.949 × 10^1^	1.60 × 10^15^	1.34 × 10^3^
*F*_6_	Ave	3.36 × 10^3^	1.601 × 10^3^	6.34 × 10^3^	1.60 × 10^3^
	Std	2.07 × 10^5^	1.601 × 10^3^	4.45 × 10^5^	1.10 × 10^1^
*F*_7_	Ave	2.36 × 10^5^	1.601 × 10^3^	2.06 × 10^7^	2.53 × 10^5^
	Std	1.79 × 10^10^	6.108 × 10^1^	7.40 × 10^13^	4.33 × 10^4^
*F*_8_	Ave	8.85 × 10^3^	2.295 × 10^3^	1.68 × 10^4^	2.31 × 10^3^
	Std	9.47 × 10^6^	2.472 × 10^1^	2.79 × 10^5^	4.63 × 10^1^
*F*_9_	Ave	3.30 × 10^3^	2.575 × 10^3^	3.80 × 10^3^	2.61 × 10^3^
	Std	1.07 × 10^4^	7.444 × 10^1^	4.87 × 10^3^	4.79 × 10^1^
*F*_10_	Ave	3.06 × 10^3^	2.897 × 10^3^	7.43 × 10^3^	2.87 × 10^3^
	Std	1.07 × 10^3^	2.338 × 10^1^	5.82 × 10^5^	1.05 × 10^1^
Decision result	+/=/−	1/3/6	3/4/3	0/0/10	--

**Table 9:** Effect of local population size *m* on the performance of the SCAEICS.

*m*	*f*_1_ (*p* = 3.3074 × 10^−76)^			*f*_3_ (*p* = 6.8459 × 10^−223^)			*f*_6_ (*p* = 1.0364 × 10^−141^)		
	Average Optimal Value	Standard Deviation	Rank	Average Optimal Value	Standard Deviation	Rank	Average Optimal Value	Standard Deviation	Rank
4	7.60 × 10^−232^	0	1	6.78 × 10^−205^	0	1	7.34 × 10^−9^	1.04 × 10^−8^	3
5	2.06 × 10^−181^	0	3	7.40 × 10^−155^	1.05 × 10^−154^	3	4.24 × 10^−11^	3.92 × 10^−11^	1
6	5.94 × 10^−219^	0	2	1.42 × 10^−203^	0	2	9.81 × 10^−9^	1.24 × 10^−8^	2
7	9.01 × 10^−170^	0	4	2.63 × 10^−141^	3.72 × 10^−141^	4	8.59 × 10^−10^	2.05 × 10^−10^	4
*m*	*f*_8_ (*p* = 1.8302 × 10^−72)^			*f*_13_ (*p* = 2.3981 × 10^−187^)			*f*_17_ (*p* = 3.1354 × 10^−278^)		
	Average Optimal Value	Standard Deviation	Rank	Average Optimal Value	Standard Deviation	Rank	Average Optimal Value	Standard Deviation	Rank
4	−12,569.4857	9.44 × 10^−4^	3	9.21 × 10^−9^	1.30 × 10^−8^	2	3.98 × 10^−1^	4.58 × 10^−7^	2
5	−12,569.4866	5.78 × 10^−4^	2	2.17 × 10^−8^	3.02 × 10^−8^	3	3.98 × 10^−1^	1.42 × 10^−4^	4
6	−12,569.4866	4.61 × 10^−4^	1	3.89 × 10^−10^	8.74 × 10^−11^	1	3.98 × 10^−1^	1.72 × 10^−7^	1
7	−12,569.4852	2.34 × 10^−3^	4	1.96 × 10^−8^	2.59 × 10^−8^	4	3.98 × 10^−1^	6.08 × 10^−8^	3
*m*	*f*_19_ (*p* = 6.4911 × 10^−198^)			*f*_21_ (*p* = 3.562 × 10^−100^)			*f*_23_ (*p* = 1.2524 × 10^−142^)		
	Average Optimal Value	Standard Deviation	Rank	Average Optimal Value	Standard Deviation	Rank	Average Optimal Value	Standard Deviation	Rank
4	−3.44	3.11 × 10^−2^	4	−10.1531	2.73 × 10^−4^	4	−10.5321	3.91 × 10^−5^	4
5	−3.68	1.69 × 10^−1^	1	−10.1531	7.06 × 10^−4^	2	−10.5363	1.73 × 10^−3^	1
6	−3.71	7.85 × 10^−2^	2	−10.1532	2.38 × 10^−4^	1	−10.5362	1.14 × 10^−3^	2
7	−3.56	2.39 × 10^−1^	3	−10.1521	1.46 × 10^−4^	3	−10.5362	9.17 × 10^−5^	3

**Table 10:** Performance comparison of different algorithms for cantilever beam optimization problems.

Algorithms	*x*_1_	*x*_2_	*x*_3_	*x*_4_	*x*_5_	*f*(*x*)
WOA	6.7223	5.6496	4.86784	2.7854	1.5343	1.7224
GWO	6.0505	5.3133	4.4703	3.5221	2.1857	1.3402
HHO	6.2829	5.2835	4.4123	3.6826	2.0938	1.3415
SSA	6.7314	4.3729	4.4344	2.9367	4.1988	1.8389
SCA	5.0881	5.2855	3.5683	3.6543	3.8544	1.4901
SCAEICS	6.9328	5.8733	4.9051	4.5246	2.4903	1.3401

**Table 11:** Comparison of the performance of different algorithms for the three-rod truss optimization problem.

Algorithms	Maximum Value	Minimum Value	Standard Deviation	*x*_1_	*x*_2_	*f* (*x*)
WOA	267.7761	263.8988	1.2117	0.7975	0.3837	265.1009
GWO	264.2903	271.0781	2.2715	0.8146	0.3410	267.6625
HHO	263.8972	265.0614	0.2509	0.7879	0.4286	264.0875
SSA	263.8973	263.9679	0.0160	0.7873	0.4121	263.9181
SCA	263.929	282.8427	6.4548	0.7935	0.3984	266.6682
SCAEICS	263.8962	263.9349	0.0075	0.7816	0.3404	263.9055

## Data Availability

The data supporting the reported results are available from the authors upon reasonable request.
